# The Effects of Sodium-Glucose Cotransporter 2-Inhibitors on Steatosis and Fibrosis in Patients with Non-Alcoholic Fatty Liver Disease or Steatohepatitis and Type 2 Diabetes: A Systematic Review of Randomized Controlled Trials

**DOI:** 10.3390/medicina59061136

**Published:** 2023-06-12

**Authors:** Ioana-Cristina Bica, Roxana Adriana Stoica, Teodor Salmen, Andrej Janež, Špela Volčanšek, Djordje Popovic, Emir Muzurovic, Manfredi Rizzo, Anca Pantea Stoian

**Affiliations:** 1 The Doctoral School of “Carol Davila”, University of Medicine and Pharmacy, 020021 Bucharest, Romania; 2 The Department of Diabetes, Nutrition and Metabolic Diseases, “Carol Davila” University of Medicine and Pharmacy, 030167 Bucharest, Romania; 3 The Department of Endocrinology, Diabetes and Metabolic Diseases, University Medical Center, The Medical Faculty, The University of Ljubljana, 1000 Ljubljana, Slovenia; 4 The Clinic for Endocrinology, Diabetes and Metabolic Disorders, The Clinical Centre of Vojvodina, The Medical Faculty, The University of Novi Sad, 21137 Novi Sad, Serbia; 5 The Department of Internal Medicine, The Endocrinology Section, The Clinical Center of Montenegro, The Faculty of Medicine, The University of Montenegro, 81000 Podgorica, Montenegro; 6 School of Medicine, Promise Department, University of Palermo, 90100 Palermo, Italy

**Keywords:** sodium-glucose cotransporter 2-inhibitors, steatosis, fibrosis, non-alcoholic fatty liver disease, non-alcoholic steatohepatitis, diabetes

## Abstract

Type 2 Diabetes Mellitus (T2DM) and non-alcoholic fatty liver disease (NAFLD) are part of metabolic syndrome and share multiple causal associations. Both conditions have an alarmingly increasing incidence and lead to multiple complications, which have an impact on a variety of organs and systems, such as the kidneys, eyes, and nervous and cardiovascular systems, or may cause metabolic disruptions. Sodium-glucose cotransporter 2-inhibitors (SGLT2-i), as an antidiabetic class with well-established cardiovascular benefits, and its class members have also been studied for their presumed effects on steatosis and fibrosis improvement in patients with NAFLD or non-alcoholic steatohepatitis (NASH). The MEDLINE and Cochrane databases were searched for randomized controlled trials examining the efficacy of SGLT2-i on the treatment of NAFLD/NASH in patients with T2DM. Of the originally identified 179 articles, 21 articles were included for final data analysis. Dapagliflozin, empagliflozin, and canagliflozin are some of the most used and studied SGLT2-i agents which have proven efficacy in treating patients with NAFLD/NASH by addressing/targeting different pathophysiological targets/mechanisms: insulin sensitivity improvement, weight loss, especially visceral fat loss, glucotoxicity, and lipotoxicity improvement or even improvement of chronic inflammation. Despite the considerable variability in study duration, sample size, and diagnostic method, the SGLT2-i agents used resulted in improvements in non-invasive markers of steatosis or even fibrosis in patients with T2DM. This systematic review offers encouraging results that place the SGLT2-i class at the top of the therapeutic arsenal for patients diagnosed with T2DM and NAFLD/NASH.

## 1. Introduction

The global diabesity epidemic is closely associated with the increasing number of all manifestations of metabolic syndrome (MetS) [1]. Type 2 Diabetes Mellitus (T2DM) is a progressive, metabolic condition with increasing incidence and a pandemic prevalence, becoming a well-known public health problem [2]. Non-alcoholic fatty liver disease (NAFLD) is a progressive multisystemic disease as well, although strictly defined as hepatic fat accumulation diagnosed through imaging or histology but with no secondary causes of this condition, such as significant alcohol consumption, the use of steatogenic therapies, and viral or hereditary etiologies [3]. The global prevalence of NAFLD was estimated to be around 25%; however, in certain metabolic conditions characterized by insulin resistance, the prevalence of NAFLD is more than double compared to the general population [4]. NAFLD and T2DM share key pathophysiological factors, including insulin resistance, glucotoxicity, lipotoxicity, low-grade chronic inflammation, and cytokine and adipokine dysregulation [5]. Therefore, NAFLD prevalence in T2DM patients is considerably higher, up to 70%, with a much worse prognosis [6]. Non-alcoholic steatohepatitis (NASH) is estimated to have a prevalence ranging from 15.9% to 68.3% of all NAFLD cases, with a higher prevalence in patients with obesity and T2DM [7]. Fibrotic progression in patients with NASH is about 40%, with an average annual progression rate of 0.09%. The presence of T2DM is one of the predictors of rapid fibrotic progression [8]. Advanced stages of NASH may evolve to hepatocellular carcinoma (HCC), with an annual HCC incidence rate of 5.29 per 1000 persons per year in NASH, while in the case of NAFLD patients, the annual HCC incidence rate is reported to be 0.44 per 1000 persons per year [4]. However, patients with non-cirrhotic NAFLD are at increased risk of extrahepatic malignancies and cardiovascular (CV) events, representing the leading cause of death [9,10,11]. Furthermore, NAFLD is independently associated with specific microvascular conditions, such as diabetic kidney disease [12]. Therefore, the treatment of NAFLD/NASH must extend beyond the treatment for liver disease [13].

Lifestyle intervention is the cornerstone therapeutic approach for NAFLD patients [14]; however, given the lack of approved pharmacological options, drug candidates repositioned from related conditions, such as T2DM, are being investigated for liver-related outcomes. Sodium-glucose cotransporter-2 inhibitors (SGLT-2i) are an antidiabetic class with established cardiovascular benefits and promising effects for T2DM and NAFLD/NASH patients [15]. SGLT-2i inhibit glucose reabsorption in the proximal tubule and increase urinary glucose excretion, thereby exhibiting a decrease in glucose levels [16]. SGLT-2i class members are known to result in a mean weight loss of 3% [17], as well as their favorable effects on diabetic kidney disease progression [18]. Several previous studies reported that canagliflozin [19], dapagliflozin [20], and empagliflozin [21], irrespective of SGLT-2 selectivity, have a beneficial effect on hepatic steatosis or fibrosis in T2DM patients with NAFLD.

**Figure 1:**
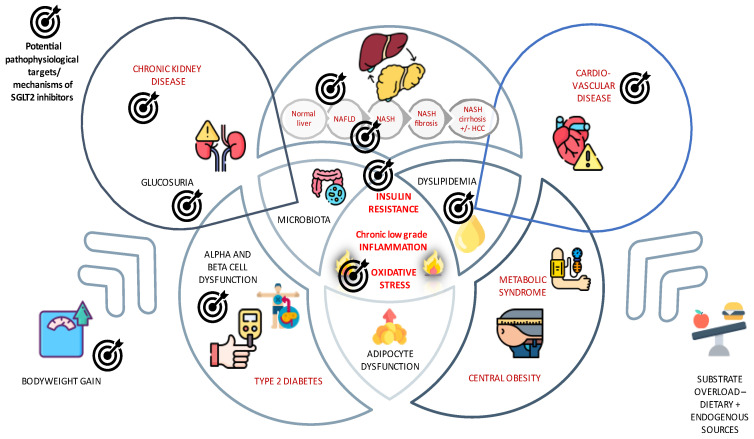
Potential pathophysiological targets/mechanisms of SGLT2 inhibitors. The interplay between NAFLD and diseases of the metabolic spectrum (Type 2 Diabetes Mellitus and other components of metabolic syndrome) addresses many potential mechanisms by which SGLT-2 inhibitors could act beyond inhibiting SGLT2-mediated renal glucose reabsorption and inducing glycosuria. Furthermore, NAFLD has been associated with an increased risk for chronic kidney disease and cardiovascular diseases, and SGLT-2i are known for their renoprotective and cardioprotective properties. Their different pathophysiological targets/mechanisms include the inhibition of de novo lipogenesis, leading to hepatic steatosis and improving oxidative and inflammatory responses, which directly and indirectly improve insulin sensitivity. Abbreviations: NAFLD: non-alcoholic fatty liver disease, NASH: non-alcoholic steatohepatitis, HCC: hepatocellular carcinoma, SGLT-2i: Sodium glucose cotransporter-2 inhibitors.

Another described mechanism in NAFLD/NASH pathogenesis is insulin resistance through hepatokines such as fetuin A, fetuin B, retinol-binding protein 4 (RBP4), and selenoprotein P [22]. Hepatokines are liver-secreted proteins, whose concentrations and effects alter the metabolic processes through endocrine signaling [22]. Some nutraceuticals have previously shown some beneficial effects on liver enzymes, oxidative stress, and inflammation markers in patients with T2DM and NAFLD, and this has been very recently reviewed in a position paper from the International Lipid Expert Panel (ILEP) [23]; in this document, the authors critically reviewed all the available evidence regarding the effect of different nutraceuticals on NAFLD-related parameters, concluding that many nutraceuticals have been studied in relation to NAFLD but none have sufficient evidence to recommend their routine use. By contrast, promising data have been reported in recent years with the use of SGLT2-i and another antihyperglycemic drug class, the Glucagon-Like Peptide-1 Receptor Agonists (GLP-1 RA), which demonstrated benefits in terms of NAFLD/NASH amelioration in patients with T2DM through weight loss, a decrease in insulin resistance, or inhibition of lipogenesis in liver cells [24,25]. SGLT2-i promise improvements in insulin sensitivity via improving glucose toxicity, caloric disposition, and weight loss, reducing the inflammatory response, reducing the oxidative stress, improving the beta cell function, and even alpha cell secretagogue effects [26]. Although extra-renal expression of SGLT-2 was described in various tissues, the expression of SGLT-2 in human hepatocytes still remains unclear [27], in contrast with GLP-1RA, with known beneficial effects on NAFLD/NASH that are partly mediated by hepatocyte GLP-1 receptors [28]. The mechanisms by which SGLT2 inhibitors can improve NAFLD and NASH are shown in Figure 1.

Despite the clear shared metabolic pathways and multiple treatment possibilities, no FDA-approved specific agents are available to specifically target NAFLD/NASH in T2DM patients. On the other hand, therapeutic inertia is still an important issue in treating patients with T2DM [29]; therefore, it is of critical importance to find the best treatment option for this category of patients with NAFLD and T2DM, and consequently greater CVD burden. Since several treatments, including nutraceuticals, have shown flaws in steatosis and fibrosis in patients with NAFLD/NASH and Type 2 Diabetes, we performed a systematic review of randomized controlled trials with the use of SGLT-2i.

## 2. Materials and Methods

We developed a reproducible protocol for our study following the recommendations of Preferred Reporting Items for Systematic Reviews and Meta-Analyses (PRISMA) for the systematic review protocol checklist [30]. Furthermore, we used the Population, Intervention, Comparison, Outcome and Study Design (PICOS) strategy to guide our study rationale and to carry out a clear, helpful, and systematic literature search. A systematic review of the literature was performed in February 2023 under the CRD42023397432 PROSPERO registration number. We searched the MEDLINE and Cochrane databases for randomized controlled trials that reported the effect of SGLT2 inhibitors on NAFLD/NASH, according to the Preferred Reporting Items for Systematic Reviews and Meta-Analyses statement for the conduction of meta-analyses of the observational studies [30].

We included in our systematic review only studies published in English, conducted on humans, with latest access on 25 February 2023. The search terms included: (fatty liver OR NASH OR non-alcoholic steatohepatitis OR NAFLD OR non-alcoholic fatty liver disease OR non-alcoholic) AND (SGLT2-i OR sodium-glucose cotransporter-2 inhibitors OR empagliflozin OR dapagliflozin OR canagliflozin OR ipragliflozin OR luseogliflozin OR tofogliflozin OR licogliflozin AND (randomized controlled-trial OR RCT) AND (type 2 diabetes mellitus OR type 2 DM OR T2DM). Two readers (ICB, TS) reviewed the title and abstract of the selected articles for the determination of inclusion as well as the full text of the selected studies. The included studies were RCTs conducted on non-pregnant adults aged 18 or above and published in peer-reviewed journals published before 25 February 2023. Other included studies were randomized, double-blind, placebo-controlled studies, or active-controlled, parallel-group studies. 

Full-text articles were eligible for inclusion if they utilized a SGLT2-inhibitor agent, included NAFLD patients, included recognized NAFLD/NASH tools for diagnosing (imaging, biopsy, and established hepatic steatosis and fibrosis scores), and provided details regarding clinical outcomes (change in hepatic steatosis and lobular inflammation, ballooning, and fibrosis) related to NAFLD. Studies evaluating other causes of liver diseases were excluded. Letters, editorials, conference abstracts, reviews, and commentaries were not included. Only the article with the most comprehensive data was included for multiple publications of the same RCT. The exclusion criteria referred to studies published in other languages than English, and which had no data on alcohol consumption or other liver diseases, such as viral hepatitis B or C.

This systematic review included 21 randomized controlled trials. We used the Newcastle–Ottawa Quality Assessment Scale to evaluate the quality of the trials and we included only studies that scored intermediate (4–6 points) or high (7–9 points) on this scale. Additionally, we used the Cochrane risk-of-bias tool (RoB2 version) to assess the quality of RCTs, and we only included low-risk bias studies.

## 3. Results

### 3.1. Literature Search and the Characteristics of the Included Studies

**Figure 2:**
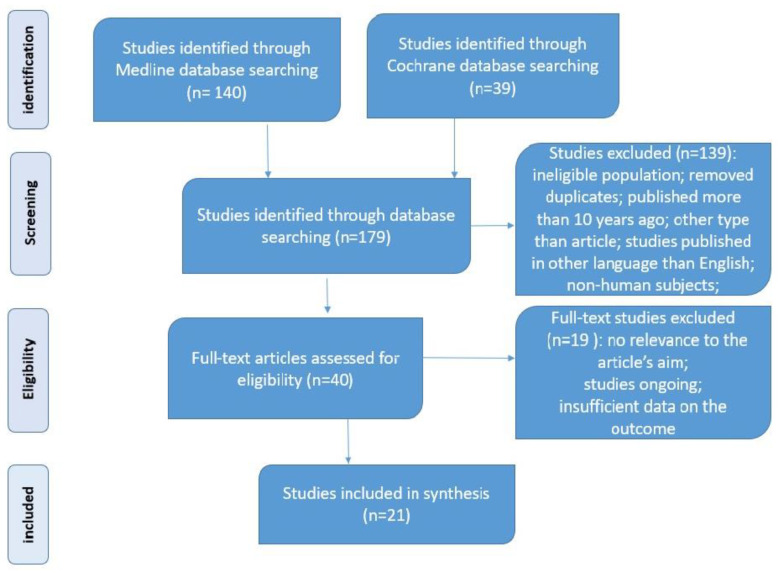
Preferred Reporting Items for Systematic Reviews and Meta-Analyses (PRISMA) flow diagram.

SGLT2 inhibitors have been studied in several randomized controlled clinical trials for their promising effects on improving NAFLD/NASH in patients with T2DM. The literature search resulted in the identification of 179 articles. A total of 40 articles were appropriate for a full review. After excluding studies without a definitive diagnosis of NAFLD with only surrogate liver outcomes, ongoing studies, and studies without relevance to the aim of this article, a total of 21 articles were included for data analysis (Figure 2).

Hepatic injury was diagnosed through different methods, from Magnetic Resonance Imaging Proton Density Fat Fraction (MRI-PDFF) to Fibro-Scan, computed tomography (CT), Positron Emission Tomography–Computed Tomography (PET-CT), bioelectrical impedance, liver proton-magnetic resonance spectroscopy (^1^H-MRS), different hepatic steatosis or fibrosis scores (FLI, NAFLD score, FIB4, NAFLD fibrosis score), or even liver biopsy.

The study periods ranged from 8 weeks [31] to 105 weeks [32] and included 15 [31] to 695 [33] patients in the study group; they were either placebo-controlled studies or parallel-group comparison trials. Trials that included standard treatment as a comparator used: pioglitazone [34], glimepiride [35], metformin [36], or other antidiabetic agents other than SGLT2-i [21].

Most studies included well-controlled T2DM patients, but two studies included patients with NAFLD, without DM, to explore the impact of this drug class on steatosis and fibrosis, independent from the benefits of improved glucose control [37,38].

The overlap in inclusion criteria of the included studies was substantial, namely: T2DM, non-pregnant adults, alcohol consumption of less than 10 g/day in women and 20 g/day in men, and DM (except “Effect of Empagliflozin on Liver Steatosis and Fibrosis in Patients With Non-Alcoholic Fatty Liver Disease Without Diabetes: A Randomized, Double-Blind, Placebo-Controlled Trial” and “Comparison between dapagliflozin and teneligliptin in non-alcoholic fatty liver disease patients without type 2 diabetes mellitus: a prospective randomized study”). The exclusion criteria included: liver disease of other etiology, including hepatitis B, hepatitis C, autoimmune hepatitis, drug-induced liver disease, alcohol consumption >20 g per day in women or >30 g in men, advanced renal insufficiency, heart failure class II-IV New York Heart Association (NYHA), and pregnancy.

The significant variability in study duration, population, and/or diagnostic tools is illustrative of the massive scientific interest and the constant need for further research on how the SGLT2-I antidiabetic class can improve hepatic steatosis and even fibrosis in this vulnerable subset of patients.

### 3.2. Dapagliflozin and NAFLD/NASH

Dapagliflozin was studied in several clinical trials and it demonstrated benefits in terms of improving NAFLD (non-alcoholic fatty liver disease) and even NASH, as presented in Table 1. 

Shimizu et al. evaluated the effect of dapagliflozin on hepatic steatosis and fibrosis, diagnosed via transient elastography (TE) in patients with T2DM and NAFLD. TE (Fibroscan) assesses the severity of liver injury using controlled attenuation parameter (CAP) and liver stiffness (LS). LS ≥ 8.0 kPa was considered indicative of significant liver fibrosis. The trial showed that the administration of dapagliflozin 5 mg/day for 24 weeks led to the improvement of liver steatosis through significant improvement in CAP in the study group, compared to the active-controlled group. Moreover, they discovered that dapagliflozin could prevent the progression of liver fibrosis in patients with T2DM and NAFLD, with baseline fibrosis (LS > 8 kPa). Furthermore, liver stiffness measurement (LSM) showed a strong positive correlation with validated liver fibrosis indexes, such as the FIB-4 index and the NAFLD fibrosis score [20].

Another team of researchers from Japan used computed tomography to assess the comparative effect of dapagliflozin (5 mg/day), glimepiride (0.5–1 mg/day), and pioglitazone (7.5–15 mg/day) on visceral fat loss and liver-to-spleen ratio after 28 weeks. The SGLT2-i agent and the thiazolidinedione had comparable effects on increasing liver-to-spleen ratio, but patients receiving dapagliflozin experienced significant weight loss and visceral fat loss compared to the ones in the glimepiride or pioglitazone groups, respectively [35].

Furthermore, Johansson et al. conducted a 52-week randomized trial in which they compared the effects of dapagliflozin (10 mg/day) plus saxagliptin (5 mg/day) as an add-on to metformin with glimepiride (1, 2, 3, 4 or 6 mg) as an add-on to metformin. Not at all surprisingly, the patients in the first group benefited from a significant reduction in liver fat (PDFF-IRM), visceral fat, and subcutaneous adipose tissue, all measured through MRI [36].

The EFFECT-II (effects of dapagliflozin and n-3 carboxylic acids on non-alcoholic fatty liver disease in individuals with type 2 diabetes) trial was a randomized placebo-controlled trial that also used MRI-PDFF to assess the liver fat content in patients with NAFLD and T2 diabetes mellitus. The effects of dapagliflozin (10 mg daily) and omega 3 carboxylic acids were investigated on liver fat content, either together or separately. After 12 weeks of treatment, the patients receiving omega 3 (4 g daily) plus dapagliflozin (10 g daily) had the best reduction in liver volume and liver fat content measured by means of PDFF-MRI. Furthermore, dapagliflozin improved fasting and 2 h plasma glucose levels, fasting insulin concentrations, and insulin sensitivity as measured by homeostasis-model-assessment-estimated insulin resistance (HOMA-IR), none of these effects being observed in the omega 3 or placebo groups [39].

Another RCT, with a duration of 8 weeks, demonstrated that dapagliflozin 10 mg/day decreased visceral fat tissue, liver volume, and liver fat content, all measured by means of MRI, and also interleukin-6 and N-terminal prohormone of brain natriuretic peptide levels. However, the study did not find any effect on tissue insulin sensitivity, measured by means of [^18^F]-fluorodeoxyglucose and positron emission tomography (PET-CT) during a hyper insulin-euglycemic clamp [31].

Other researchers used validated scores and indices based on metabolic and hepatic parameters to diagnose and evaluate the outcome, such as fatty liver index (FLI), the Fibrosis-4 (FIB-4) index, non-alcoholic fatty liver disease (NAFLD) score, or non-alcoholic fatty liver disease fibrosis score (NFS) [33,34]. Gastaldelli et al. demonstrated that the combination of dapagliflozin 10 mg on a daily basis and exenatide 2 mg once a week was more efficient at reducing non-alcoholic steatosis and fibrosis measured by FLI, FIB-4, NAFLD score, and NFS in patients with T2DM than dapagliflozin plus placebo or exenatide plus placebo [33].

On the other hand, EXENDA (The Effects of Combined Dapagliflozin and Exenatide Versus Dapagliflozin and Placebo on Ectopic Lipids in Patients with Uncontrolled Type 2 Diabetes Mellitus) was a randomized controlled trial that demonstrated no additional effects on the reduction of hepatocellular lipids despite better glycemic control in patients with T2DM and NAFLD treated with a combination of dapagliflozin 10 mg on a daily basis and exenatide 2 mg once a week. Unlike the previously mentioned study, this trial was a single-center study with a small population size. MRS was used for diagnosis and follow-up, in addition to validated scores such as FLI, FIB-4, or NFS [40].

A team of researchers from Thailand studied the effect of dapagliflozin 10 mg on a daily basis compared to placebo in reducing intrahepatic lipid content in patients with T2DM. Using computed tomography (CT), they found that patients in the active group had significant reductions in body weight, body fat, and visceral fat/subcutaneous fat ratio, as well in hemoglobin A1c (HbA1c) and alanine aminotransferase (ALT), with no difference in adiponectin, leptin, and tumor necrosis factor (TNF-alfa) levels after 12 weeks of treatment [41].

A phase 3 liver fat MRI understudy of dapagliflozin and saxagliptin (10/5 mg) versus glimepiride (1–6 mg) demonstrated favorable effects on liver fat in addition to improved glucose control and metabolic variables [32].

### 3.3. Empagliflozin and NAFLD/NASH

The E-LIFT (The effects of Empagliflozin on Liver Fat in Patients With Type 2 Diabetes and Non-alcoholic Fatty Liver Disease) trial was one of the relevant RCTs that studied empagliflozin’s effects on liver fat in patients with T2DM. The 50 included patients were randomly assigned to either the empagliflozin group (10 mg daily) or the control one (standard treatment, without empagliflozin) for 20 weeks. The NAFLD-related outcomes were assessed by means of MRI-PDFF. The patients in the empagliflozin group experienced significant reductions in liver fat, despite similar glycemic control [21], as seen in Table 2.

A similar RCT, conducted by Kahl et al., demonstrated the effect of empagliflozin (25 mg daily) in reducing liver fat content, measured by means of MRI, promoting weight loss and increasing adiponectin levels, but without improvement in insulin sensitivity [42].

The EMPACEF (The effects of empagliflozin on ectopic fat stores and myocardial energetics in type 2 diabetes) study searched for a beneficial effect of empagliflozin 10 mg on a daily basis for 12 weeks in terms of ectopic fat in high-risk patients with T2DM. The authors discovered that the SGLT2-i agent could decrease liver fat content, but not epicardial adipose tissue (an important metabolic active fat tissue), as they used MRI to measure visceral fat [43].

This is in line with the recent findings of Chehrehgosha et al., who demonstrated in their RCT that empagliflozin 10 mg improved the CAP score and LSM compared to pioglitazone, while significant body weight and abdominal visceral fat reductions were observed only in the empagliflozin group, while both increased in the placebo and pioglitazone groups [44].

The impact of the most frequently studied SGLT-2i empagliflozin 25 mg was compared to ursodeoxycholic acid 250 mg and/or placebo in decreasing liver fat content (LFC). In this study, the use of empagliflozin was associated with improvement in hepatic steatosis as measured by MRI-PDFF [45] at 24 weeks.

### 3.4. Canagliflozin and NAFLD/NASH

Cusi et al. investigated the effect of canagliflozin on hepatic triglyceride content and glucose metabolism in patients with T2DM. In this randomized placebo-controlled group, patients receiving canagliflozin 300 mg daily for 24 weeks experienced an improvement in hepatic insulin sensitivity, along with a decrease in intrahepatic triglyceride content (measured by means of MRS) proportionate to the magnitude of weight loss, compared to the placebo group [46], as seen in Table 3.

### 3.5. Other SGLT2-i and NAFLD/NASH

Other SGLT2-i agents, such as ipragliflozin (50 mg) and luseogliflozin (2.5 mg), demonstrated significant effects in reducing liver fat deposition, measured as liver-to-spleen attenuation ratio on CT, in patients with T2DM [47,48,49]. Both agents were studied for 6 months, with pioglitazone and metformin as comparators and also exerted beneficial effects in terms of weight loss and glycemic control [48,49]. Furthermore, the additive effect of ipragliflozin 50 mg in well-controlled T2DM patients treated with metformin and pioglitazone was demonstrated as reduced hepatic fat content, measured as FLI, CAP, and NAFLD liver fat score. Ipragliflozin add-on therapy also reduced whole-body and abdominal visceral fat [50], as seen in Table 4.

Takahashi et al. investigated the effect of ipragliflozin on hepatic outcomes in patients with NAFLD and T2DM. The RCT included 55 patients with NAFLD (diagnosed through liver biopsy) and T2DM. Patients received treatment with ipragliflozin 50 mg on a daily basis of the standard of care treatment (except SGLT-2i, GLP-1 RA, or pioglitazone). More patients in the ipragliflozin group obtained a reduction in ballooning and fibrosis (52.4% vs. 24%, respectively; 57.1% vs. 16%) after 72 weeks of treatment [51].

ToPiND study is an RCT that evaluated the effects of 20 mg tofogliflozin compared to pioglitazone 15–30 mg in patients with T2DM and NAFLD. After 6 months of treatment, patients in both the SGLT2-i group and the thiazolidinedione one had a significant decrease in hepatic steatosis, appreciated by means of MRI-PDFF, with no difference in the FIB-4 index and type IV collagen between baseline and post-treatment in both groups. Interestingly enough, there were no differences between the groups regarding the relative change in hepatic steatosis. Even though tofogliflozin-treated patients experienced body weight loss and the pioglitazone ones had gained weight, all of them had similar changes in terms of hepatic steatosis. Another strength of this trial is the PNLPLA3 and TM6SF2 genotyping use. The researchers did not find any significant difference in allele frequency of PNPLA3 and TM6SF2, respectively, in both groups and the efficacy of the treatment did not undergo any variations in both groups after the stratification dependent on PNLPLA3 and TM6SF2 genotype [52], as it can be observed in Table 5.

In a 48-week RCT, tofogliflozin 20 mg on a daily basis led to the improvement of hepatocellular ballooning (*p* = 0.002), and lobular inflammation (*p* = 0.003) in 40 T2DM patients with NAFLD, evaluated by means of liver biopsy. Moreover, changes in energy metabolism, inflammation, and fibrosis were correlated with hepatic gene expression in the tofogliflozin group, compared to the glimepiride (0.5 mg daily) group [53].

### 3.6. New Perspectives

Licogliflozin, leading to the inhibition of both sodium-glucose cotransporters 1 and 2, was tested in 107 patients with phenotypic or histologic NASH. After 12 weeks of treatment with licogliflozin 150 mg, they showed a significant 32% placebo-adjusted reduction in serum ALT [54]. The occurrence of diarrhea in 77% of the patients and the fact that the lower 30 mg dose did not reach this primary endpoint make licogliflozin a candidate for combination treatment.

## 4. Discussion

Regardless of the established cardiovascular benefits that the SGLT-2i class is known for, there are some precaution measures for this antidiabetic class that clinicians must take into account.

One of the most acknowledged side effects when using SGLT-2i therapy is genitourinary infection [55]. Nevertheless, a recent Korean study demonstrated that genital and urinary tract infections appear to be two- to three-fold more frequent in females and patients over 60 years old [56], emphasizing the need for the rigorous monitoring of these categories.

Another concern for this class of therapy has been acute kidney injury (AKI) after initiation [57], but recent data prove that SGLT-2i class is safe and presents substantial renal protection in patients with T2DM [58,59]. Moreover, canagliflozin has demonstrated significant effects in reducing blood pressure, attributed to the osmotic diuresis this therapy produces [60]. However, the blood pressure reduction may represent an adverse effect in patients with already low intravascular volume, leading to hypotension, with a risk of unsafe falls [61] or representing a considerable factor in SGLT-2i-induced AKI through renal hypoperfusion [57,60].

Another possible adverse event (AE) that was discussed is the risk of ketoacidosis, but the data from a meta-analysis of RCTs prove that SGLT-2i did not increase the risk of ketoacidosis in patients treated with an SGLT-2i agent, compared to other antidiabetic classes [62].

A serious, but rare AE for this class is the risk of bone fracture in patients with T2DM treated with canagliflozin [63]. The CANVAS study revealed that patients treated with canagliflozin had a slightly greater risk of fracture (HR, 1.26; 95% CI, 1.04 to 1.52) than the placebo-treated ones [64]. 

Clinicians should reflect on the balance between risk and benefits, as well as strictly monitor vulnerable patients, who have been demonstrated to be at risk for these AEs.

## 5. Conclusions

The SGLT2-i class has proven benefits in reducing the risk of cardiovascular events and chronic renal impairment in patients with T2DM [65]. Knowing that NAFLD/NASH has been associated with greater risks for CV and chronic kidney disease, it seems that this drug class may address several treatment targets in individuals with T2DM and NAFLD/NASH. Furthermore, the importance of using SGLT2i for patients with NAFLD/NASH and T2DM is reinforced by mechanism research attributing the SGLT-2i class alleviation of insulin resistance, the hallmark of NAFLD [10,66,67]. 

Hopefully, future, more comprehensive, clinical trials will assess the efficacy, safety, and tolerability of drug candidates and lead to the approval of new pharmaceuticals for NAFLD that can be used in T2DM patients. Taking into consideration the natural evolution of NAFLD, upcoming research should clarify what stage is critical for the treatment initiation, for how long the treatment should be followed for the achievement of the best results, what the optimal dosage is for every agent, and, finally, which agent is the most appropriate for this condition. Further research is needed to clarify whether the proposed beneficial effects of SGLT-2 inhibitors are direct or mediated by body weight reduction and improved glucose control. The promise of their indirect disease-modifying action is being investigated in clinical trials including non-diabetic subjects. Overall, SGLT-2i are effective in managing glycemic control and cardio-renal risks; however, histological analyses are needed to evaluate their role in NAFLD/NASH. Furthermore, head-to-head comparison studies will be needed to consolidate individual SGLT-2i class member benefits.

For the moment, we still have lifestyle measures at our fingertips, one of the least expensive therapeutic interventions [14,68] and the cornerstone of the treatment in patients with NAFLD/NASH with or without T2DM. The present review offers insights for clinicians working with T2DM patients with NAFLD with the hope that it will provide them with the best available treatment option to mitigate their increased cardiovascular risk and possibly offer liver-related benefits.

## Figures and Tables

**Table 1:** The effects of dapagliflozin 5 mg/day on NAFLD/NASH in patients with T2DM.

Author	Design/Comparator	Number of Patients	Study Duration	Method of Diagnosis	Outcome	Measure Method
Shimizu et al., 2019 [20]	RCT/placebo	57	24 weeks	FibroScan	Improvement of hepatic steatosis and fibrosis	CAP and LSM
Kinoshita et al., 2020 [35]	RCT/pioglitazone or glimepiride	98	28 weeks	CT	Improvement of NAFLD	Liver-to-spleen ratio
Johansson et al., 2020 [36]	RCT/glimepiride plus placebo	444/59 in the MRI substudy	52 weeks	MRI-PDFF	Improvement of NAFLD	Liver fat percentage and adipose tissue volumes
EFFECT-II/Eriksson et al., 2018 [39]	RCT/omega-3 carboxylic acids/ placebo	83	12 weeks	MRI-PDFF + PNPLA3 polymorphism	Improvement of NAFLD	Liver fat content
Rasku et al., 2019 [31]	RCT/placebo	32	8 weeks	MRI-PDFF	Improvement of NAFLD	Liver fat content
Gastaldelli et al., 2020 [33]	RCT/exenatide combination/placebo combination	695	52 weeks	FLI, FIB-4, NAFLD fibrosis score	Improvement in non-invasive steatosis and fibrosis score	Non-invasive scores
EXENDA/Harreiter et al., 2021 [40]	RCT/exenatide combination/placebo	30	24 weeks	MRS + FLI + FIB-4	Improvement in intrahepatic lipid content	HCL
Phrueksotsai et al., 2021 [41]	RCT/placebo	38	12 weeks	CT	Changes in intrahepatic lipid contents	Liver attenuation index
Frias et al., 2022 [32]	RCT	338	105 weeks	MRI	Changes in adipose tissue and liver fat	MRI

MRI-PDFF = Magnetic Resonance Imaging Proton Density Fat Fraction; MRS = magnetic resonance spectroscopy; CT = computed tomography; CAP = controlled attenuation parameter; LSM = liver stiffness measurement; EXENDA = The effects of Combined Dapagliflozin and Exenatide Versus Dapagliflozin and Placebo on Ectopic Lipids in Patients With Uncontrolled Type 2 Diabetes Mellitus; EFFECT-II = Effects of dapagliflozin and n-3 carboxylic acids on non-alcoholic fatty liver disease in people with type 2 diabetes; HCL = Hepato-cellular lipid.

**Table 2:** The effects of empagliflozin 10 mg/day on NAFLD/NASH in patients with T2DM.

Author	Design/Comparator	Number of Patients	Study Duration	Method of Diagnosis	Outcome	Measure Method
E-LIFT trial (Kuchay et al., 2018) [21]	RCT/Standard treatment	50	20 weeks	MRI-PDFF	Liver fat content < 6%	Liver fat content
Kahl et al., 2020 [42]	RCT/placebo	84	24 weeks	^1^H-MRS	Change in liver fat content	Liver fat content
EMPACEF(Gaborit et al., 2021) [43]	RCT/placebo	56	12 weeks	^1^H-MRS	Changes in liver, pancreatic, and myocardial trygliceride content	Epicardial fat volume; intrahepatic and pancreatic fat content
Chehrehgosha et al., (2021) [44]	RCT/placebo	78	24 weeks	FibroScan	Improvement of hepatic steatosis and fibrosis	CAP and LSM
Elhini et al., (25 mg/day 2022) [45]	RCT/UDCA	240	24 weeks	MRI-PDFF	Change in liver fat content	Liver fat content

^1^H-MRS = Proton magnetic resonance spectroscopy; UDCA = ursodeoxycholic acid; CAP = Controlled attenuation parameter; LSM = liver stiffness measurement; MRI-PDFF = Magnetic Resonance Imaging–Proton Density Fat Fraction; RCT = randomized controlled trial.

**Table 3:** The effects of canagliflozin 300 mg/day on NAFLD/NASH in patients with T2DM.

Author	Design/Comparator	Number of Patients	Study Duration	Method of Diagnosis	Outcome	Measure Method
Cusi et al., 2019 [46]	RCT/placebo	56	24 weeks	^1^H-MRS	Reduction in intrahepatic TG	Intrahepatic TG accumulation

RCT = randomized controlled trial; TG = triglyceride.

**Table 4:** Effects of ipragliflozin 50 mg/day on NAFLD/NASH in patients with T2DM.

Author	Design/Comparator	Number of Patients	Study Duration	Method of Diagnosis	Outcome	Measure Method
Takahashi et al., 2022 [51]	RCT/standard treatment	55	72 weeks	Liver biopsy	Reduction in intrahepatic ballooning and fibrosis	Ballooning and fibrosis
Ito et al., 2017 [48]	RCT/pioglitazone	66	24 weeks	CT	Change in L/S ratio	L/S ratio
Han et al., 2020 [50]	RCT/metformin + pioglitazone	45	24 weeks	FibroScan CT	Improvement in CAP; reduction in VFA	CAP, VFA

L/S ratio = liver-to-spleen attenuation ratio; VFA = abdominal visceral fat area; CAP = controlled attenuation parameter; RCT = randomized controlled trial.

**Table 5:** The effects of other SGLT-2i on NAFLD/NASH in patients with T2DM.

Author	Design/Comparator	Number of Patients	Study Duration	Method of Diagnosis	Outcome	Measure Method
ToPiND study (tofogliflozin 20 mg/day, 2021) [52]	RCT/pioglitazone	40	24 weeks	MRI-PDFF	Change in MRI-PDFF	Hepatic fat fraction on MRI-PDFF
Takeshita et al., (tofogliflozin 20 mg/day, 2022) [53]	RCT/glimepiride	40	48 weeks	Liver biopsy; FibroScan	Change in hepatocellular ballooning, inflammation, and lobular fibrosis	CAP; LSM; hepatocellular ballooning, inflammation, and lobular fibrosis
Shibuya et al., (luseogliflozin 2.5 mg/day, 2017) [49]	RCT/metformin	32	6 months	CT	Change in L/S ratio	L/S ratio

## Data Availability

Not applicable.
